# Differences in Eating Quality Attributes between Japonica Rice from the Northeast Region and Semiglutinous Japonica Rice from the Yangtze River Delta of China

**DOI:** 10.3390/foods10112770

**Published:** 2021-11-11

**Authors:** Ying Zhu, Dong Xu, Zhongtao Ma, Xinyi Chen, Mingyue Zhang, Chao Zhang, Guodong Liu, Haiyan Wei, Hongcheng Zhang

**Affiliations:** Jiangsu Co-Innovation Center for Modern Production Technology of Grain Crops, Jiangsu Key Laboratory of Crop Genetics and Physiology, Yangzhou University, Yangzhou 225009, China; dx120180066@yzu.edu.cn (Y.Z.); dx120180065@yzu.edu.cn (D.X.); mazhongtao1106@163.com (Z.M.); cxy2215502977@163.com (X.C.); mingyue_z04@163.com (M.Z.); zc312539195@163.com (C.Z.); guodongliu@yzu.edu.cn (G.L.); hywei@yzu.edu.cn (H.W.)

**Keywords:** japonica rice, eating quality characteristics, cooked rice texture, chemical compositions, starch fine structure, starch physicochemical properties

## Abstract

Differences in cooked rice and starch and protein physicochemical properties of three japonica rice were compared systematically. Cultivars of japonica rice, Daohuaxiang2, from Northeast China (NR) and two semiglutinous japonica rice (SGJR), Nangeng46 and Nangeng2728, from the Yangtze River Delta (YRD) were investigated. Both Daohuaxiang2 and Nangeng46 achieved high taste values, but there were great differences in starch and protein physicochemical properties. Daohuaxiang2 showed higher apparent amylose content (AAC), lower protein content (PC), and longer amylopectin (especially fb2 and fb3) and amylose chain lengths, resulting in thicker starch lamellae and larger starch granule size. Its cooked rice absorbed more water and expanded to larger sizes. All of these differences created a more compact gel network and harder but more elastic cooked rice for Daohuaxiang2. Nangeng46 produced a lower AAC, a higher PC, shorter amylopectin and amylose chain lengths, thinner starch lamellae, and smaller starch granule sizes, creating a looser gel network and softer cooked rice. The two SGJR, Nangeng46 and Nangeng2728, had similar low AACs but great differences in taste values. The better-tasting Nangeng46 had a lower PC (especially glutelin content) and higher proportion of amylopectin fa chains, which likely reduced the hardness, improved the appearance, and increased the adhesiveness of its cooked rice. Overall, both types of japonica rice from the NR and YRD could potentially have good eating qualities where Nangeng46’s cooked rice was comparable to that of Daohuaxiang2 because of its lower AC. Moreover, its lower PC and higher proportion of amylopectin fa chains likely improved its eating quality over the inferior-tasting SGJR, Nangeng2728. This research lays a foundation for the improvement of the taste of japonica rice in rice breeding.

## 1. Introduction

China is the largest japonica rice (*Oryza sativa* L. ssp. japonica) producer in the world, accounting for the largest planting area and the highest yield of japonica rice [1,2,3]. The two major japonica rice-growing regions in China, the Northeast Region (NR) and Yangtze River Delta (YRD), account for more than 80% of the japonica rice production [4]. With the rise of living standards in China, dietary structures have begun to change and a preference for high quality rice with good palatability has grown among rice consumers [5]. Rice breeders need to select varieties for traits related to preferred eating qualities.

Local environmental factors such as temperature throughout rice cultivation can affect traits related to eating quality. For example, low average daily temperature during the grain-filling stage of rice and significant differences in day and night temperatures [6] results in low protein content (PC) [7], amylose content (AC) of 15–20% [8], and high pasting viscosity [1] in japonica rice grown in China’s NR. These desired characteristics reduce the hardness, increase the adhesiveness, and improve the sensory quality of cooked rice of grains produced in this region [9,10]. The higher quality of japonica rice from the NR than from the YRD [11] has increased the popularity of NR rice over YRD rice according to consumers.

Rice paddies in the YRD produced the highest yields of japonica rice in China due to large amounts of nitrogen fertilizer applied during rice growth [12]. However, greater inputs of nitrogen fertilizer elevate PCs in rice, which increases the hardness and consequently diminishes the eating quality of cooked rice [13]. The greater inputs of nitrogen fertilizer along with the frequent occurrence of high temperatures and high humidity conditions during grain filling in the YRD [14] decreased the appearance of rice grains. These constraints of poor grain appearance [15] and poor eating quality [16] restrict the market competitiveness of rice grown in the YRD. 

With advancements in plant breeding technology, cultivars of semiglutinous japonica rice (SGJR) containing relatively low ACs (5–15%) were developed [17,18]; these cultivars greatly improved the eating quality of the japonica rice from the YRD [19]. Some SGJR cultivars from YRD, such as “Nangeng46” and “Nangeng9108”, won high praise in rice taste competitions many times in China and Japan, and the tastes of which were comparable to the japonica rice from the NR. Consequently, breeders have paid much more attention to SGJR, and many cultivars of SGJR are gradually becoming more commonly grown in large areas in the YRD. Many studies on SGJR have reported on some important characteristics of its starch, such as small particle size [20], high relative crystallinity [21], and high swelling power [18]. Other studies found that a low AC in SGJR with good taste quality reduced the gelatinization temperature [22] and retrogradation degree [18], increased softness of the cooked rice, and improved the cooking and eating qualities of SGJR [17].

The taste of the conventional japonica rice from NR and SGJR from YRD are similarly preferred by consumers currently [9]. Are there any differences in other eating quality characteristics between the japonica rice from the two different regions? Do all SGJR cultivars grown in the YRD have high quality in taste? Are there any differences between the different SGJR cultivars? To find answers to these questions, we compared traits from three cultivars of rice: (1) Daohuaxiang2, the most prominent high-quality japonica rice from the NR; (2) Nangeng46, the most representative SGJR from the YRD; and (3) Nangeng2728, a cultivar of SGJR from the YRD with a less desirable taste than that of the other two cultivars. The differences in cooked rice properties, flour/paste properties, chemical compositions, and starch physicochemical properties of the three japonica rice were compared systematically. The main objectives of this study were: (1) to elucidate differences in eating quality characteristics between the two good-tasting japonica rice from the NR and YRD and (2) to determine the underlying causes of potential differences in eating quality characteristics between the two SGJR cultivars. This research lays a foundation for the improvement of taste when breeding japonica rice.

## 2. Materials and Methods

### 2.1. Materials

Daohuaxiang2 was provided by Wuchang Rice Research Institute (44° 92′ N, 127° 15′ E, Heilongjiang, China). This area belongs to the continental monsoon climate in a mid-latitude. The annual average temperature is about 4 °C and the annual precipitation is about 625 mm. Nangeng46 was provided by Changshu Agricultural Science Research Institute (31°65′ N, 120°75′ E, Suzhou, China). This area belongs to a humid subtropical monsoon climate with mild climate, four distinct seasons and abundant rainfall. The annual average temperature is about 15.7 °C and the annual precipitation is about 1100 mm. Nangeng2728 was provided by Huaiyin Agricultural Science Research Institute (33°63′ N, 119°03′ E, Huai’an, China). This area belongs to a typical transitional monsoon climate in the north subtropical north warm temperate zone. The annual average temperature is about 14 °C and the annual precipitation is about 960 mm.

### 2.2. Rice Taste Value

The taste value was determined by a taste analyzer (STA1A, SATAKE, Japan) using the preset selection of “Japanese japonica rice” for the detection line and following the method described by Zhang et al. [23] with minor modifications. The taste analyzer measures the appearance of rice and other indexes by optical principle, and it synthesizes these parameters to obtain a taste value from a sample. A sample of 30 g of polished grains from each cultivar was placed into a stainless steel tank, washed with running purified water for 30 s, drained, and reconstituted with purified water to bring the ratio of rice to water to 1:1.33. The sample was then soaked for 30 min in the tank, covered with filter paper, and sealed with a rubber ring. The stainless steel tank was placed into an electric rice cooker (JT783, Midea, Shunde, China), covered, steamed for 30 min, and kept warm for 10 min. The tank was taken out from the rice cooker, and the steamed rice was gently stirred and turned over. Then, the tank was covered with filter paper again and cooled for 20 min using a supporting air-cooling device. Afterward, the filter paper was replaced with the supporting steel cover, and the steamed rice was sealed and allowed to cool at room temperature (25 °C) for 90 min. An 8-g sample of steamed rice was placed into a stainless steel ring (30-mm diameter and 9-mm height) and then pressed the rice into a cake. The rice cake was placed in a measuring tank, and the rice taste analyzer was inserted to measure appearance and taste value of the steamed rice sample. Three rice cakes were measured for each steamed rice sample, and each face (top and bottom) of each cake was measured once.

### 2.3. Rice Texture Profile Analysis

Rice hardness, springiness, stickiness, and balance value were obtained using a TA.XT-Plus Texture analyzer with a P/36R probe attachment (Stable Micro Systems Ltd., Surrey, UK) and following the method described by Wang et al. [24]. The texture profile analysis simulates the experience of human mastication of rice through these specific parameters. Samples were prepared and cooked under the same conditions as described above. We also took a single grain of cooked rice from the upper, middle, and lower sections of the stainless steel tank of cooked rice and then placed the three grains of rice together on the base plate of the analyzer. The probe was set to descend at 1 mm/s, returned, and then the compression cycle was repeated. All textural analyses of each sample were replicated six times and results presented are mean values.

### 2.4. Rice Cooking Properties

The water absorption rate and volume expansion rate of rice, iodine blue value, and weight of dried solids of rice slurry were followed using the method described by Zhan et al. [25] with minor modifications. A 35-g sample of head-milled rice was placed into a stainless steel strainer after measuring its volume by the drainage method. Then, the rice was washed with tap water by panning the rice four times, then rinsing once with distilled water, and finally putting each washed sample into a 1-L beaker filled with 500 mL distilled water before heating. After the water boiled for 20 min, the strainer was vertically taken off and held above the beaker of boiled liquid until any liquid from the strainer stopped dripping. The weight and volume of the cooked rice were measured. We then poured the remaining rice slurry from the beaker into a 1-L volumetric flask, allowed it to cool to room temperature, and diluted it with distilled water to 1 L before blending and returning the blended slurry into the original beaker. Fifty milliliters of the blended slurry was pipetted into a 100-mL beaker, placed in an oven at 70 °C for 24 h, and then weighed to calculate the weight of dried solids. Ten milliliters of the blended slurry was centrifuged for 10 min at 8000 r/min, and 1 mL of the supernatant was pipetted into a 100-mL volumetric flask. We transferred 5 mL of 0.5-mol/L hydrochloric acid and 1 mL of 0.2 mol/L I_2_-KI solution into the flask before diluting the mixture with distilled water to the full 100 mL. The mixture sat for 20 min prior to taking colorimetric measurements at 660 nm. The water absorption rate of rice = (weight of cooked rice (g))/(weight of head-milled rice (g)) × 100%, the volume expansion rate of rice = (volume of cooked rice (mL))/(volume of head-milled rice (mL)) × 100%, the weight of dried solids of rice slurry = (weight of dried solids (mg))/(weight of head-milled rice (g)) × (1000 (mL))/(50 (mL)), and the iodine blue value of rice slurry was expressed as an absorbance value.

### 2.5. Flour Preparation and Starch Isolation

The milled rice was ground in a mill (FOSS 1093 Cyclotec Sample Mill, Hoganas, Sweden) and then sieved through a 100-mesh screen. Starch was isolated according to the method of Hu et al. [21].

### 2.6. Rice Flour Pasting Properties

Pasting properties of rice flour were determined using a Rapid Visco-Analyzer (RVA TecMaster, Perten, Sweden) and following the method described by Ma et al. [9]. Rice flour (3 g) was weighed and placed into an RVA sample canister and approximately 25 mL of distilled water was added so the moisture content of the rice flour totaled 12%, and then the sample canister was transferred into the RVA for testing. The entire length of the program cycle was 13 min. Starch samples cycled through a heating–cooling program starting at 50 °C for 1 min, then increasing the heat from 50 to 95 °C at a rate of 12 °C /min, holding at 95 °C for 2.5 min, cooling to 50 °C at 12 °C /min, and ending at another hold at 50 °C for 2 min. The pasting parameters of peak viscosity (PV), trough viscosity (TV), breakdown (BD), final viscosity (FV), and setback (BD) were recorded for analysis.

### 2.7. Rice Flour Rheological Properties

The freshly prepared gels of rice flour samples obtained from the RVA machine were used to obtain data on their dynamic viscoelastic properties as measured by a rheometer (Discovery HR-2, TA Instruments, New Castle, DE, USA) following the method described by Tangsrianugul et al. [26] with minor modifications and 40-mm-diameter parallel metal plates with a gap of 1000 μm, which was equilibrated at 25 °C. To obtain the dynamic viscoelastic measurement, the linear viscoelastic range was determined with strain sweep (0.01–100%) at a fixed frequency of 1 rad/s. After that, a dynamic frequency sweep was conducted by applying a constant strain of 5%, which was within the linear region, over a frequency range of 0.1–100 rad/s. The mechanical spectra were obtained by recording the dynamic moduli G′, G″ and tan δ as a function of frequency. The G′ value is a measure of the dynamic elastic or storage modulus related to the material’s response as a solid. The G″ value is a measure of the dynamic viscous or loss modulus related to the material’s response as a fluid. The tan δ value is the loss tangent defined as the ratio of G″ to G′.

### 2.8. Starch Composition

Apparent amylose content (AAC) in rice flour was measured using the iodine colorimetric method of Tan et al. [27]. The total starch content in rice flour was determined using a total starch assay kit (Megazyme, Bray, Ireland) following the protocol described in the kit’s operation manual. The following equation was used to obtain the content of amylopectin: amylopectin content = total starch content-AAC.

### 2.9. Isolation and Determination of Protein Components

The albumin, globulin, prolamin, and glutelin were extracted by ultrapure water, 5% NaCl solution, 70% ethyl alcohol solution, and 0.2% NaOH solution, respectively, following the method described by Zheng et al. [28] with minor modifications. Rice flour (0.2 g) in a 10-mL centrifuge tube was oscillated with 2 mL ultrapure water for 2 min before centrifuging at 3000 r/min for 15 min, and the supernatant which contained the albumin was collected. To fully extract as much of the remaining albumin as possible from the precipitate, the precipitate was mixed with 2 mL ultrapure water, oscillated, and centrifuged to collect the supernatant three times. The first extraction of albumin and any remaining extracted albumin were pooled together. The precipitate remaining after the albumin extraction was mixed with 2 mL 5% NaCl solution to extract globulin by following the same process used to collect the albumin. The precipitate, after completing the globulin extraction, was mixed with 2 mL 70% ethyl alcohol solution, oscillated for 2 min, then oscillated in an 80 °C water bath for 30 min with two small glass beads in a sealed tube, and then oscillated for two more minutes before centrifuging at 3000 r/min for 15 min to obtain the supernatant containing prolamin. This prolamin extraction process was repeated three times to extract any remaining prolamin. The precipitate remaining after the prolamin extraction was mixed with 2 mL 0.2% NaOH solution to extract the glutelin by following the same process used to collect the albumin and globulin. For each cultivar’s sample of rice flour, we determined its protein components using an automatic Kjeldahl apparatus (Kjeltec 8400, Foss, Denmark). Because the apparatus measures only the content of elemental nitrogen rather than the contents of proteins, the results were multiplied by 5.95 to obtain the contents of total protein and the four protein components of interest.

### 2.10. Starch Molecular Size-Distributions

The molecular size distributions of whole amylose molecules were analyzed using an Agilent 1100 series system (PSS, Mainz, Germany) equipped with a refractive index detector (RI; ShimadzuRID-10A, Shimadzu Corp., Kyoto, Japan) as described by Wang et al. [29]. Samples were separated using a GRAM precolumn and GRAM 30 and 3000 analytical columns (PSS) at a flow rate of 0.3 mL min^−1^. The molecular size distribution of debranched amylose were characterized using the same analytical size exclusion chromatography setup after debranching using isoamylase. Size separation of debranched starch was performed using a GRAM precolumn and GRAM 100 and 1000 analytical columns (PSS) at a flow rate of 0.6 mL min^−1^. Pullulan standards with peak molecular weights ranging from 342–2.35 × 10^6^ were used for calibration.

### 2.11. Amylopectin Fine Structure Analysis

The fine structure of amylopectin was investigated utilizing fluorophore-assisted carbohydrate electrophoresis (FACE) as described by Li et al. [16]. First, freeze-dried debranched starch was labeled using 8-aminopyrene-1, 3, 6, trisulfonic acid (APTS) as described by Wang et al. [30]. Then, the separation of APTS-labeled debranched starch molecules was undertaken using a PA-800 Plus (Beckman-Coulter, CA, USA) FACE system equipped with an N–CHO-coated capillary and coupled with a solid-state laser-induced fluorescence detector. The carbohydrate separation buffer was used at 25 °C at a setting of 30 kV.

### 2.12. Particle Size Analysis

The particle size distribution of starch was investigated using a laser diffraction particle size analyzer (Mastersizer 2000, Malvern, UK). About 0.2 g starch samples were immersed in 500 mL absolute ethyl alcohol and stirred at 2000 rpm. The instrument was adjusted to measure starch particle sizes ranging from 0.1 to 2000 μm.

### 2.13. X-ray Diffraction Analysis

X-ray diffractograms were obtained using an X-ray powder diffractometer (D8 Advance, Bruker-AXS, Karlsruhe, Germany) operated at 200 mA and 40 kV, over a diffraction angle (2θ) range of 3–40°, with a step size of 0.02° and a sampling interval of 0.6 s. Relative crystallinity (%) was calculated with the MDI Jade 6 software.

### 2.14. Small-Angle X-ray Scattering (SAXS) Analysis

The lamellar structure of starch was analyzed using a small-angle X-ray scattering instrument (Bruker NanoStar, Vantec 2000, Bruker, Germany) following the method of Yuryev et al. [31]. The SAXS data were analyzed using DIFFRACplus NanoFit software, and SAXS spectrum parameters were determined following a simple graphical method [32].

### 2.15. Determination of Thermal Properties

The thermal properties were determined using a differential scanning calorimetry (DSC) analyzer (Model 200 F3 Maia, Netzsch, Germany) according to Lu & Lu [33]. Five milligrams of starch was mixed with water at two times the weight of the starch and then sealed in an aluminum pan at 4 °C overnight. The DSC analyzer was first calibrated using a standard pan (empty pan) as reference and then heated from 20 °C to 100 °C at a rate of 10 °C /min. Onset temperature (T_o_), peak temperature (T_p_), conclusion temperature (T_c_), and gelatinization enthalpy (ΔHg) were calculated by the TA Universal Analysis 2000 software. The samples were heated from 20 °C to 100 °C at a rate of 10 °C /min and then stored at 4 °C for 7 days to determine the retrogradation properties. Retrogradation enthalpy (ΔHr) was calculated by the TA Universal Analysis 2000 software, and retrogradation percentage (%R) = ΔHr/ΔHg × 100%.

### 2.16. Statistical Analysis

In characterizing the various parameters of rice quality, at least two replicate measurements were obtained unless otherwise specified. All data shown are means of biological repeats from three plots with standard deviations (SD). For experiments with multiple comparisons, the data were analyzed by one-way analysis of variance (ANOVA) with the least significant difference (LSD) test. Differences were determined to be statistically significant where *p* < 0.05.

## 3. Results

### 3.1. Cooked Rice Properties

The taste values of Daohuaxiang2 and Nangeng46 were significantly higher than those of Nangeng 2728 (Table 1). The ranking of cultivars by the hardness of their cooked rice was Daohuaxiang2 > Nangeng2728 > Nangeng46. The hardness of Daohuaxiang2 cooked rice was 42.36% and 23.49% higher than that of Nangeng46 and Nangeng2728, respectively. The elasticity of cooked rice for each cultivar ranked as follows: Daohuaxiang2 > Nangeng46 > Nangeng2728. The elasticity of Daohuaxiang2 was 15.07% and 19.45% higher than that of Nangeng46 and Nangeng2728, respectively. The water absorption rate and volume expansion rate of cooked rice and iodine blue value of the rice soup showed the same trend as that of the elasticity of cooked rice. The values of appearance and adhesiveness of cooked rice were both highest for Nangeng46 followed by those for Daohuaxiang2 and then those for Nangeng2728. Nangeng46′s appearance was 6.49% and 41.38% higher, respectively, and adhesiveness was 2.06% and 2.76% higher, respectively, than those of Daohuaxiang2 and Nangeng2728. The weight of dried solids of the rice soup showed the same trend as that of appearance and adhesiveness.

### 3.2. Rice Flour and Paste Properties

**Figure 1:**
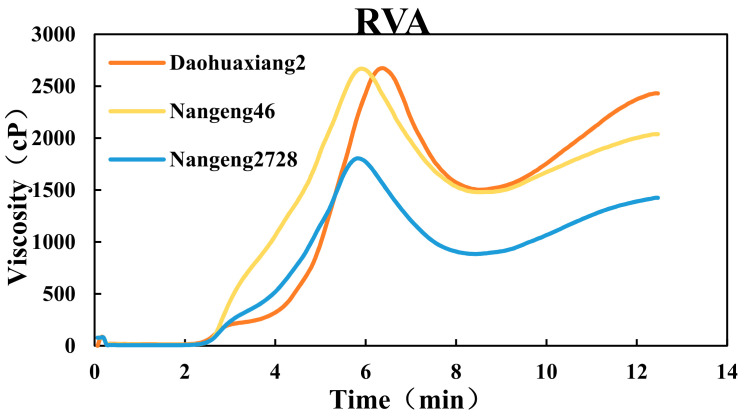
RVA profile of different japonica rice.

During pasting, the highest to lowest values obtained for peak viscosity, trough viscosity and final viscosity of rice flour ranked the cultivars as Daohuaxiang2 > Nangeng46 > Nangeng2728 (Figure 1). The breakdown ranked the cultivars as Nangeng46 > Daohuaxiang2 > Nangeng2728 (Table 2), where the breakdown of Nangeng46 was 1.54% and 29.13% higher than that of Daohuaxiang2 and Nangeng2728, respectively. The setback values of Daohuaxiang2 were highest followed by Nangeng2728 and then Nangeng46. The setback of Daohuaxiang2 was 61.59% and 36.15% higher than that of Nangeng46 and Nangeng2728, respectively. The pattern of consistence values was similar to the trends of the three first-order viscosity indexes, and the consistence of Daohuaxiang2 was 66.31% and 71.53% higher than that of Nangeng46 and Nangeng2728, respectively.

**Figure 2:**
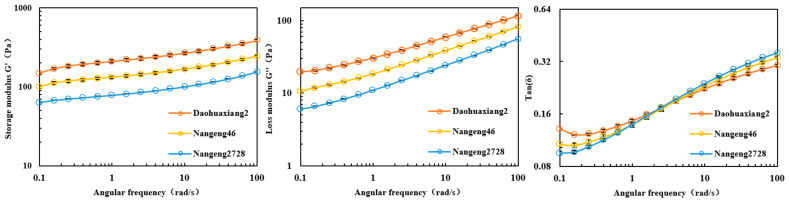
Rheological properties of different japonica rice.

The rheological properties of rice paste were measured at 25 °C after pasting (Figure 2). In the linear viscoelastic region and with the increase of angular frequency, the storage modulus G′, loss modulus G″, and tan value gradually increased. During the entire dynamic frequency scanning process, both the storage modulus G′ and loss modulus G″ values ranked the cultivars as follows: Daohuaxiang2 > Nangeng46 > Nangeng2728. The Tan value was highest in Daohuaxiang2, moderate in Nangeng46, and lowest in Nangeng2728 at the low-frequency scanning stage (angular frequency < 1 rad/s), while the value was highest in Nangeng2728, moderate in Nangeng46, and lowest in Daohuaxiang2 at the high-frequency scanning stage (angular frequency > 10 rad/s).

### 3.3. Compositions of Starch and Protein

The contents of total starch and apparent amylose in the cultivars ranked the cultivars in the following order: Daohuaxiang2 > Nangeng46 > Nangeng2728 (Table 3). The total starch content of Daohuaxiang2 was 3.28% and 4.76% higher than that of Nangeng46 and Nangeng2728, respectively, and the apparent amylose content was 56.31% and 70.81% higher than that of Nangeng46 and Nangeng2728, respectively. The ranking of cultivars by amylopectin content was Nangeng46 > Nangeng2728 > Daohuaxiang2. The amylopectin content of Nangeng46 was 3.55% and 0.53% higher than that of Daohuaxiang2 and Nangeng2728, respectively. The hierarchical orders of cultivars based on highest to lowest contents of total protein and glutelin were both Nangeng2728 > Nangeng46 > Daohuaxiang2. The contents of total protein and of the four types of protein components (Table 3) of Daohuaxiang2 were significantly lower than those of Nangeng46 and Nangeng2728. There were no significant differences in contents of albumin, globulin, and prolamin between Nangeng46 and Nangeng2728.

### 3.4. Fine Structure of Starch

The molecular weights of total starch, amylose, and amylopectin of Daohuaxiang2 were significantly higher than those of Nangeng46 and Nangeng2728 (Table 4). The molecular weight of total starch for Dahuaxiang2 was 57.28% and 76.88% higher, the amylose molecular weight was 9.69% and 9.31% higher, and the amylopectin molecular weight was 72.78% and 89.70% higher than those of Nangeng46 and Nangeng2728, respectively. There was no significant difference in amylose molecular weight between Nangeng46 and Nangeng2728.

The 100 < X ≤ 1000 amylose chain proportion of Dahuaxiang2 was 35.14% and 35.14% higher than that of Nangeng46 and Nangeng2728, respectively. There was no significant difference in 100 < X ≤ 1000 amylose chain length proportion between Nangeng46 and Nangeng2728. The 1000 < X ≤ 2000 and 2000 < X ≤ 20000 amylose chain proportions from highest to lowest among the cultivars were Daohuaxiang2 > Nangeng46 > Nangeng2728. The 1000 < X ≤ 2000 amylose chain proportion of Daohuaxiang2 was 81.76% and 149.19% higher and the 2000 < X ≤ 20000 amylose chain proportion was 64.26% and 139.27% higher than that of Nangeng46 and Nangeng2728, respectively.

The fa chain (DP6–12) proportion of amylopectin was highest in Nangeng46, then in Daohuaxiang2, and lowest in Nangeng2728. The fa chain (DP6–12) proportion of amylopectin of Nangeng46 was 8.89% and 24.96% higher than that of Daohuaxiang2 and Nangeng2728, respectively. The fb1 chain (DP13–24) proportion of amylopectin among cultivars ranked as follows: Nangeng2728 > Nangeng46 > Daohuaxiang2. The fb1 chain (DP13–24) proportion of amylopectin of Nangeng2728 was 12.08% and 9.28% higher than that of Daohuaxiang2 and Nangeng46, respectively. The fb2 chain (DP25–36) proportion of amylopectin of Daohuaxiang2 was 2.32% and 2.32% higher than that of Nangeng46 and Nangeng2728, respectively. There was no significant difference in fb2 chain (DP25–36) proportion of amylopectin between Nangeng46 and Nangeng2728. The fb3 chain (DP37–100) proportion and average chain length of amylopectin placed the cultivars in the following order: Daohuaxiang2 > Nangeng2728 > Nangeng46. The fb3 chain (DP37–100) proportion of Daohuaxiang2 was 26.57% and 10.07% higher and the average chain length was 8.54% and 2.48% higher than those of Nangeng46 and Nangeng2728, respectively.

### 3.5. Physicochemical Properties of Starch

**Figure 3:**
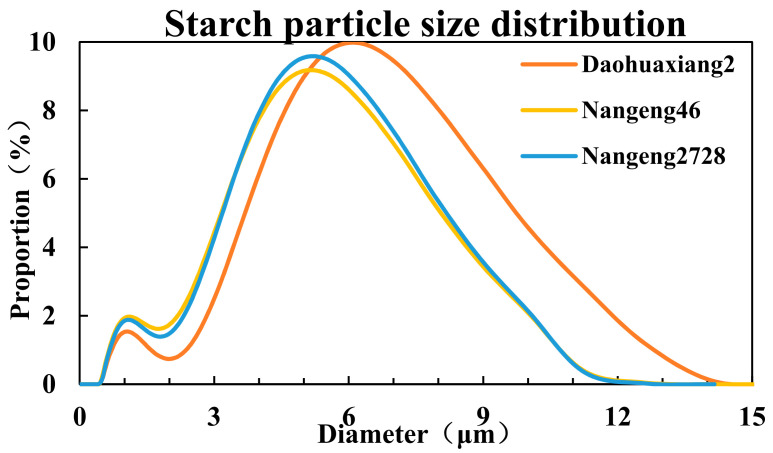
Starch particle size distribution of different japonica rice.

Nangeng46 produced the highest proportion of small starch particles (0–3 μm), while Daohuaxiang2 produced the highest proportion of large starch particles (>6 μm) (Figure 3). The average volume and average surface area of starch particles were greatest in Daohuaxiang2, moderate in Nangeng2728, and lowest in Nangeng46 (Table 5). The average volume of particles in Daohuaxiang2 was 22.88% and 20.16% higher than that in Nangeng46 and Nangeng2728, respectively, and average surface area of particles was 24.77% and 20.12% higher, respectively. The proportions of 0–2-μm diameter and 2–4-μm diameter particles placed the cultivars in the following order: Nangeng46 > Nangeng2728 > Daohuaxiang2. The order of cultivars by the proportion of 4–6-μm diameter particles was Nangeng2728 > Daohuaxiang2 > Nangeng46. From highest to lowest proportions of the 6–8-μm diameter and 8–14-μm diameter particles, the cultivars ranked as follows: Daohuaxiang2 > Nangeng2728 > Nangeng46.

Cultivar rankings by peak intensity and relative crystallinity of starch were Nangeng2728 > Nangeng46 > Daohuaxiang2 (Table 6). The peak intensity of Nangeng2728 was 143.44% and 20.42% higher than that of Daohuaxiang2 and Nangeng46, respectively, and the relative crystallinity was 24.37% and 3.78% higher, respectively. The lamellar thickness of starch put the cultivars in the order of Daohuaxiang2 > Nangeng46 > Nangeng2728, where thickness of Daohuaxiang2 was 3.63% and 8.15% higher than that of Nangeng46 and Nangeng2728, respectively.

The greatest to lowest values of each, T_o_, T_p_, T_c_, and ΔHg, ranked the three cultivars as Nangeng2728 > Nangeng46 > Daohuaxiang2. When comparing values of Nangeng2728 to those of Daohuaxiang2 and Nangeng46, T_o_ was 6.61% and 6.25% higher, T_p_ was 21.01% and 20.25% higher, T_c_ was 22.75% and 19.13% higher, and ΔHg was 75.52% and 33.73% higher, respectively. The regeneration degree of starch was tested after 7 days, and the retrogradation enthalpy (ΔHr) and retrogradation percentage (%R) placed the cultivars in the following order: Nangeng2728 > Nangeng46 > Daohuaxiang2. The ΔHr of Nangeng 2728 was 118.91% and 206.83% higher than that of Daohuaxinag2 and Nangeng 46, respectively, and the %R was 24.59% and 129.11% higher than that of Daohuaxinag2 and Nangeng 46, respectively.

## 4. Discussion

### 4.1. Mechanisms Underlying a Good Quality of Taste of Japonica Rice from the NR and YRD in China

Japonica rice grown in the NR and YRD, such as Daohuaxiang2 and Nangeng46, respectively, had excellent quality of taste (Table 1) and is popular with consumers [4]. The taste quality of cooked rice is a complex trait attributed to numerous factors [34,35]. Starch and protein are two major components in the endosperm of rice, the physicochemical properties of which affect the taste quality of cooked rice. Starch is a branched glucose polymer that usually is composed of two types of molecules: amylose and amylopectin [36]. Amylopectin chains are packed into a crystalline lattice within the lamellae of a starch granule and form crystalline layers [37]. Long amylopectin chains (especially fb2 and fb3 chains) could increases the thickness of the lamellae by passing from the crystalline region into the amorphous region of the lamellae [38,39]. When they pass among the lamellae, they increase the size of the starch granule [40]. During rice cooking, a larger starch granule size can result in greater absorption of water and swelling to a larger volume, which influences the viscoelastic properties of rice [41]. Large amounts of amylopectin and small amounts of amylose that dissolve from rice grains [42,43] gradually cover the surfaces of rice grains and form a thin film [44]. This film plays a crucial role in the appearance and adhesiveness of cooked rice [45].

As the second most abundant constituent of milled rice after starch, proteins will increase the hardness of cooked rice as past studies have reported [46,47]. Additionally, proteins have been suggested to play a role in rice quality by interacting with starch. Proteins (especially glutelin) often inhibit starch granules from absorbing water and swelling, which affects the elasticity of cooked rice. Proteins are more likely to combine with amylose during recrystallization, which results in more protein-amylose complexes filling the pores of the gel network, thus increasing the hardness of the gel and the cooked rice [48,49]. In our study, Daohuaxiang2 showed both longer lengths of amylopectin (especially fb2 and fb3 chain lengths) and amylose chains than Nangeng46, resulting in thicker starch lamellae, larger starch granule sizes [40], and a firmer double helix structure, which ultimately creates a more compact gel network and harder cooked rice [8,50]. Furthermore, the lower PC in Daohuaxiang2 than in Nangeng46 led to a smaller effect of proteins inhibiting starch granules from absorbing water and swelling during cooking. Thus, the cooked rice of Daohuaxiang2 absorbed more water and expanded to larger sizes than that of Nangeng46 (Table 1). Due to these factors, Daohuaxiang2 showed higher elasticity in its cooked rice, which improved its taste [25,51]. Despite the fact that proteins increase the hardness of cooked rice and that Nangeng46 had a higher PC than Daohuaxiang2, the gel network and cooked rice of Nangeng46 was softer than that of Daohuaxiang2 (Figure 2, Table 1). This was probably due to significant differences in AC and chain lengths of amylose and amylopectin between the two types of japonica rice. High AC and long chain lengths of amylose and amylopectin thus played a decisive role in the hardness of the gel network and cooked rice, regardless of PC. As a result, the cooked rice of Daohuaxiang2 was harder and the cooked rice of Nangeng46 was softer in texture.

### 4.2. Differences in Eating Quality Characteristics between Two SGJR Cultivars

Relatively low AC and a soft-texture in cooked rice are the most noticeable features reported of SGJR [19]. Although Nangeng46 and Nangeng2728 produced similarly low ACs (Table 3), there were great differences in cooked rice properties between the two SGJR cultivars. Of rice with similar ACs, proteins have been shown to play an important role in the hardness of cooked rice [52]. Thus, the higher PC of Nangeng2728 increased the hardness of its cooked rice compared to that of Nangeng46 (Figure 2). Consistence measured by RVA and %R determined by DSC can both be used to indicate the retrogradation of starch. In our study, the results showed that consistence of Nangeng46 was higher than that of Nangeng2728 (Table 2), while %R of Nangeng46 was lower than that of Nangeng2728 (Table 5). In fact, the consistence measured by RVA reflected the short-term retrogradation of starch, which is mainly related to the rapid recrystallization of amylose [8]. The %R determined by DSC reflected the long-term retrogradation of starch, which not only was related to the rapid recrystallization of amylose, but also to the recrystallization of amylopectin [53]. Nangeng2728 had a lower measure of consistence than Nangeng46 due to the former’s lower content and shorter chain length of amylose. The %R of Nangeng2728 was larger than that of Nangeng46 due to the larger proportion of long chain lengths of amylopectin [54]. The recrystallization of amylopectin increased gel hardness and further increased the hardness of cooked rice. In addition, Nangeng46 had a larger proportion of amylopectin fa chains than Nangeng2728. A recent study reported that short chain lengths of amylopectin are easier to dissolve than long chain lengths of amylopectin [55]. Moreover, Nangeng46 had a higher iodine blue value for its rice soup, indicating that more amylose was dissolved while cooking this rice [45]. Consequently, Nangeng46 had a higher dry matter weight of rice soup, which was attributed to the greater amounts of dissolved amylopectin and amylose. The dissolution of these two types of starch thickened the film on the surface of cooked rice grains and ultimately improved the appearance and increased the adhesiveness of the cooked rice.

## 5. Conclusions

Starch and protein physicochemical properties are of great necessity in improving the taste of japonica rice in future. The critical point lies in the fact of achieving a balance of hardness, softness, adhesiveness and elasticity of cooked rice.

Nangeng46 and Nangeng2728, the two SGJR that are cultivated in the YRD and had lower ACs than the AC of rice from the NR, presented great differences in eating quality between them. Reducing PC (especially glutelin content) and increasing the proportion of amylopectin fa chains can improve the eating quality of SGJR from the YRD. Between the two types of YRD rice, Nangeng46, with its lower PCs (especially glutelin content) and higher proportion of amylopectin fa chains, is the better rice based on appearance and adhesiveness, lower hardness and higher taste value.

Japonica rice produced in both the NR and YRD can have good eating qualities. Daohuaxiang2, the more widely-preferred japonica rice that is produced in the NR, exhibited harder but more elastic cooked rice. Nangeng46, a SGJR from the YRD, exhibited a softer texture of cooked rice because of its lower AC. Therefore, as long as a balance of hardness, softness, adhesiveness and elasticity is achieved in cultivars of rice, the cooked grains are expected to have excellent taste irrespective of the amylose and protein contents and other physicochemical properties.

## Figures and Tables

**Table 1:** Differences of cooked rice properties among different japonica rice.

Cultivar	Taste Analyzer Properties		Texture Properties			Cooking Properties			
	Taste Value	Appearance	Hardness (g)	Elasticity (%)	Adhesiveness (g)	Water Absorption Rate (%)	Volume Expansion Rate (%)	Iodine Blue Value (A)	Weight of Dried Solids (mg/g)
Daohuaxiang2	80 ± 1 ab	7.7 ± 0.1 b	205 ± 4 a	0.565 ± 0.003 a	1167 ± 1 b	391 ± 4 a	532 ± 3 a	1.404 ± 0.004 a	186 ± 1 b
Nangeng46	82 ± 1 a	8.2 ± 0.1 a	144 ± 2 c	0.491 ± 0.001 b	1191 ± 1 a	354 ± 3 b	447 ± 4 b	0.284 ± 0.004 b	222 ± 1 a
Nangeng2728	62 ± 1 c	5.8 ± 0.1 c	166 ± 1 b	0.473 ± 0.001 c	1159 ± 2 c	297 ± 2 c	375 ± 3 c	0.254 ± 0.003 c	154 ± 1 c

Data are expressed as the mean ± SD (*n* = 3). Values for each index within a column followed by different lowercase letters are significantly different at the 5% probability level.

**Table 2:** Differences of rice flour pasting properties among different japonica rice.

Cultivar	Peak Viscosity (cP)	Trough Viscosity (cP)	Final Viscosity (cP)	Breakdown (cP)	Setback (cP)	Consistence (cP)
Daohuaxiang2	2673 ± 4 a	1503 ± 3 a	2431 ± 4 a	1170 ± 1 b	−242 ± 1 a	928 ± 1 a
Nangeng46	2668 ± 7 a	1480 ± 5 b	2038 ± 7 b	1188 ± 2 a	−630 ± 1 c	558 ± 2 b
Nangeng2728	1804 ± 4 b	884 ± 4 c	1425 ± 6 c	920 ± 1 c	−379 ± 2 b	541 ± 2 c

Data are expressed as the mean ± SD (*n* = 3). Values for each index within a column followed by different lowercase letters are significantly different at the 5% probability level.

**Table 3:** Differences of chemical components among different japonica rice.

Cultivar	Starch Components (%)			Protein Components (%)				
	Total Starch Content	AAC	Amylopectin Content	Total Protein Content	Albumin Content	Globulin Content	Prolamin Content	Glutelin Content
Daohuaxiang2	88.92 ± 0.06 a	15.10 ± 0.11 a	73.80 ± 0.17 c	6.39 ± 0.06 c	0.49 ± 0.01 b	0.64 ± 0.03 b	0.96 ± 0.02 b	3.44 ± 0.04 c
Nangeng46	86.10 ± 0.07 b	9.66 ± 0.09 b	76.42 ± 0.05 a	7.21 ± 0.05 b	0.58 ± 0.03 a	0.80 ± 0.02 a	1.09 ± 0.01 a	3.62 ± 0.05 b
Nangeng2728	84.88 ± 0.04 c	8.84 ± 0.10 c	76.02 ± 0.05 b	7.84 ± 0.06 a	0.56 ± 0.01 a	0.76 ± 0.02 a	1.12 ± 0.02 a	4.33 ± 0.03 a

Data are expressed as the mean ± SD (*n* = 3). Values for each index within a column followed by different lowercase letters are significantly different at the 5% probability level. AAC, apparent amylose content.

**Table 4:** Differences of molecular weight and chain length distribution of starch among different japonica rice.

Cultivar	Molecular Weight (Da)			Chain Length Proportion of Amylose (%)			Chain Length Proportion of Amylopectin (%)				
	Total Starch	Amylose	Amylopectin	100 < X ≤ 1000	1000 < X ≤ 2000	2000 < X ≤ 20,000	Fa (DP6–12)	Fb1 (DP13–24)	Fb2 (DP25–36)	Fb3 (DP37–100)	Average Chain Length
Daohuaxiang2	3493860 ± 9468 a	183319 ± 566 a	5341014 ± 5658 a	6.23 ± 0.11 a	3.09 ± 0.03 a	5.24 ± 0.05 a	28.00 ± 0.04 b	44.12 ± 0.01 c	11.92 ± 0.03 a	15.96 ± 0.03 a	21.87 ± 0.04 a
Nangeng46	2221393 ± 7797 b	167127 ± 366 b	3091176 ± 5284 b	4.61 ± 0.03 b	1.70 ± 0.02 b	3.19 ± 0.02 b	30.49 ± 0.02 a	45.25 ± 0.03 b	11.65 ± 0.02 b	12.61 ± 0.03 c	20.15 ± 0.04 c
Nangeng2728	1975251 ± 6772 c	167713 ± 713 b	2815500 ± 5335 c	4.61 ± 0.01 b	1.24 ± 0.04 c	2.19 ± 0.02 c	24.40 ± 0.01 c	49.45 ± 0.01 a	11.65 ± 0.01 b	14.50 ± 0.01 b	21.34 ± 0.01 b

Data are expressed as the mean ± SD (*n* = 3). Values for each index within a column followed by different lowercase letters are significantly different at the 5% probability level.

**Table 5:** Differences of starch particle size distribution among different japonica rice.

Cultivar	Average Volume	Average Surface Area	0~2 μm	2~4 μm	4~6 μm	6~8 μm	8~14 μm
Daohuaxiang2	5.853 ± 0.001 a	3.707 ± 0.001 a	11.45 ± 0.01 c	20.15 ± 0.01 c	27.27 ± 0.01 b	26.84 ± 0.01 a	14.34 ± 0.01 a
Nangeng46	4.763 ± 0.001 c	2.971 ± 0.001 c	18.98 ± 0.01 a	27.94 ± 0.01 a	26.85 ± 0.01 c	20.20 ± 0.01 c	6.03 ± 0.01 c
Nangeng2728	4.871 ± 0.001 b	3.086 ± 0.001 b	17.41 ± 0.01 b	27.27 ± 0.02 b	27.97 ± 0.01 a	21.19 ± 0.01 b	6.16 ± 0.01 b

Data are expressed as the mean ± SD (*n* = 3). Values for each index within a column followed by different lowercase letters are significantly different at the 5% probability level.

**Table 6:** Differences of starch crystal structure and thermal properties among different japonica rice.

Cultivar	Crystal Structure of Starch			Thermal Properties					
	Peak Intensity (Counts)	Lamellar Thickness (nm)	Relative Crystallinity (%)	T_o_ (°C)	T_p_ (°C)	T_c_ (°C)	ΔHg (J/g)	ΔHr (J/g)	%R (%)
Daohuaxiang2	87.7 ± 1.3 c	9.42 ± 0.02 a	28.68 ± 0.15 c	59.0 ± 0.2 b	63.3 ± 0.1 c	69.0 ± 0.1 c	9.60 ± 0.04 c	3.49 ± 0.02 b	36.39 ± 0.13 b
Nangeng46	177.3 ± 0.1 b	9.09 ± 0.05 b	34.37 ± 0.28 b	59.2 ± 0.1 b	63.7 ± 0.1 b	71.1 ± 0.1 b	12.60 ± 0.12 b	2.49 ± 0.02 c	19.79 ± 0.02 c
Nangeng2728	213.5 ± 1.1 a	8.71 ± 0.01 c	35.67 ± 0.07 a	62.9 ± 0.1 a	76.6 ± 0.1 a	84.7 ± 0.2 a	16.85 ± 0.07 a	7.64 ± 0.02 a	45.34 ± 0.29 a

Data are expressed as the mean ± SD (*n* = 3). Values for each index within a column followed by different lowercase letters are significantly different at the 5% probability level. T_o_, onset temperature; T_p_, peak temperature; T_c_, conclusion temperature; ΔHg, gelatinization enthalpy; ΔHr, retrogradation enthalpy; %R, retrogradation percentage.

## Data Availability

Not applicable.
